# Supplementary material for: Associations of modern initial antiretroviral therapy regimens with all-cause mortality in people living with HIV in resource-limited settings: a retrospective multicenter cohort study in China
Source: Nat Commun. 2023 Sep 2;14:5334. doi: 10.1038/s41467-023-41051-w (PMC10475132; doi:10.1038/s41467-023-41051-w)
Supplement: Supplementary file 1 — Supplementary Information [file 41467_2023_41051_MOESM1_ESM.pdf]

Table S1 Poisson regression model for the incidence of ART switch, LTFU, CD4 improvement, all-cause mortality, AIDS-related mortality, and non-AIDS-related mortality among people living with HIV on ART

	ART switch		LTFU		CD4 improvement		All-cause mortality		AIDS-related mortality		Non-AIDS-related mortality	
	IRR (95% CI)	p-value	IRR (95% CI)	p-value	IRR (95% CI)	p-value	IRR (95% CI)	p-value	IRR (95% CI)	p-value	IRR (95% CI)	p-value
NVP	Ref.		Ref.		Ref.		Ref.		Ref.		Ref.	
EFV	0.45(0.32-0.62)	<0.001	0.72(0.42-1.22)	0.217	1.46(1.21-1.76)	<0.001	0.44(0.22-0.85)	0.015	0.32(0.31-0.33)	<0.001	0.99(0.96-1.02)	0.439
DTG	2.07(1.56-2.75)	<0.001	1.24(0.69-2.25)	0.473	1.96(1.58-2.42)	<0.001	0.23(0.07-0.74)	0.014	0.10(0.09-0.10)	<0.001	0.78(0.71-0.87)	<0.001
LPV	0.57(0.41-0.79)	0.001	0.74(0.43-1.28)	0.285	1.72(1.42-2.08)	<0.001	0.23(0.09-0.55)	0.001	0.09(0.08-0.09)	<0.001	0.87(0.85-0.89)	<0.001
Others	2.62(1.91-3.59)	<0.001	0.56(0.23-1.42)	0.223	1.77(1.39-2.26)	<0.001	0.47(0.17-1.34)	0.156	0.17(0.16-0.18)	<0.001	1.80(1.71-1.90)	<0.001

ART, antiretroviral therapy. LTFU, loss to follow-up. CD4 improvement= transition of people living with HIV from CD4 <500 cells/μL to CD4 >500 cells/μL. NVP, nevirapine. EFV, efavirenz. DTG, dolutegravir. LPV, lopinavir. Others= darunavir, raltegravie, elvitegravir and rilpivirine. IRRs, incidence rate ratios. 2-sided no adjustment for multiple comparisons.

Table S2 Poisson regression model for the incidence of ART switch, LTFU, and CD4 improvement among people living with HIV on ART, adjusting for the main variables

Variable	ART switch		LTFU		CD4 improvement	
	aIRR (95% CI)	p-value	aIRR (95% CI)	p-value	aIRR (95% CI)	p-value
Age group						
18-24	Ref.		Ref.		Ref.	
25-34	1.07(0.94-1.22)	0.293	0.89(0.73-1.1)	0.287	0.98(0.93-1.04)	0.592
35-49	0.98(0.86-1.12)	0.732	0.79(0.64-0.98)	0.031	0.92(0.86-0.98)	0.007
50+	0.81(0.7-0.94)	0.005	0.85(0.68-1.06)	0.152	0.79(0.74-0.85)	<0.001
Sex						
Male	Ref.		Ref.		Ref.	
Female	1.26(1.12-1.43)	<0.001	1.04(0.89-1.23)	0.613	1.02(0.96-1.08)	0.615
Route of transmission						
Heterosexual	Ref.		Ref.		Ref.	
Homosexual	1.07(0.95-1.2)	0.245	0.81(0.67-0.98)	0.026	0.98(0.93-1.03)	0.401
Others	1.01(0.88-1.15)	0.929	1.77(1.47-2.14)	<0.001	0.96(0.89-1.03)	0.214
Region						
Northern China	Ref.		Ref.		Ref.	
Northeastern China	0.99(0.86-1.14)	0.892	0.64(0.46-0.88)	0.007	1.1(1.03-1.18)	0.007
Southern China	1.24(1.08-1.43)	0.003	0.48(0.35-0.65)	<0.001	1.12(1.04-1.21)	0.003
Southwestern China	0.49(0.43-0.56)	<0.001	1.98(1.58-2.46)	<0.001	0.69(0.64-0.73)	<0.001
Eastern China	0.67(0.58-0.76)	<0.001	0.77(0.6-1)	0.047	0.93(0.88-0.99)	0.034
Time to ART initiation						
Same-day	Ref.		Ref.		Ref.	

1-7 days	0.97(0.77-1.22)	0.777	1.02(0.71-1.47)	0.912	0.84(0.76-0.94)	0.002
8-30 days	0.93(0.75-1.16)	0.541	0.98(0.69-1.37)	0.894	0.83(0.75-0.92)	<0.001
>30 days	0.8(0.65-1)	0.048	1.22(0.87-1.7)	0.257	0.79(0.71-0.87)	<0.001
ART year						
2017	Ref.		Ref.		Ref.	
2018	0.59(0.53-0.65)	<0.001	0.44(0.36-0.54)	<0.001	0.9(0.86-0.94)	<0.001
2019	0.27(0.23-0.32)	<0.001	0.06(0.03-0.11)	<0.001	0.78(0.73-0.83)	<0.001
ART regimen						
NVP	Ref.		Ref.		Ref.	
EFV	0.43(0.36-0.51)	<0.001	0.74(0.56-0.99)	0.042	0.98(0.88-1.1)	0.763
DTG	1.08(0.86-1.35)	0.513	1.4(0.85-2.29)	0.183	1.41(1.2-1.64)	<0.001
LPV	0.42(0.34-0.52)	<0.001	0.86(0.6-1.24)	0.427	1.3(1.14-1.48)	<0.001
Others	1.06(0.82-1.37)	0.648	0.73(0.32-1.64)	0.441	1.37(1.14-1.65)	0.001
CD4 at ART initiation (per 100)	0.79(0.77-0.81)	<0.001	1.02(0.98-1.07)	0.289	1.25(1.23-1.27)	<0.001
NRTI backbone						
3TC+TDF	Ref.		Ref.		Ref.	
3TC+AZT	1.1(0.96-1.26)	0.179	1.17(0.95-1.46)	0.147	1.02(0.95-1.09)	0.581
Others	0.78(0.63-0.96)	0.021	1.74(1.17-2.59)	0.006	1.06(0.94-1.19)	0.372

ART, antiretroviral therapy. LTFU, loss to follow-up. CD4 improvement= transition of people living with HIV from CD4 <500 cells/μL to CD4 >500 cells/μL. NVP, nevirapine. EFV, efavirenz. DTG, dolutegravir. LPV, lopinavir. Others= darunavir, raltegravie, elvitegravir and rilpivirine. aIRRs, adjusted incidence rate ratios. NRTI, nucleotide reverse transcriptase inhibitor. 3TC, lamividine. TDF, tenofovir disproxil fumarate. AZT, zidovudine. Poisson regression model with a time offset and robust variances was adjusted for main variables, including baseline age, sex, route of HIV acquisition, region, time to ART initiation, year of ART initiation, CD4+ T-cell counts, and NRTI backbone. 2-sided no adjustment for multiple comparisons.

Table S3 Cox model for the ART switch, LTFU, CD4 improvement, all-cause mortality, AIDS-related mortality, and non-AIDS-related mortality among people living with HIV on ART, adjusting for the main variables

Variable	ART switch		LTFU		CD4 improvement		All-cause mortality		AIDS-related mortality		Non-AIDS-related mortality	
	aHR (95% CI)	p-value	aHR (95% CI)	p-value	aHR (95% CI)	p-value	aHR (95% CI)	p-value	aHR (95% CI)	p-value	aHR (95% CI)	p-value
Age group												
18-24	Ref.		Ref.		Ref.		Ref.		Ref.		Ref.	
25-34	1.06(0.94-1.20)	0.336	0.89(0.73-1.09)	0.275	1.02(0.97-1.07)	0.48	1.40(0.70-2.80)	0.348	3.20(0.62-16.61)	0.176	0.96(0.43-2.16)	0.918
35-49	0.95(0.84-1.08)	0.47	0.80(0.65-0.98)	0.033	0.89(0.84-0.94)	<0.001	2.98(1.55-5.74)	0.001	6.37(2.26-18.72)	0.002	1.66(0.78-3.56)	0.19
50+	0.78(0.67-0.90)	0.001	0.89(0.72-1.10)	0.285	0.73(0.69-0.77)	<0.001	7.60(4.00-14.46)	<0.001	10.34(3.58-34.61)	<0.001	3.58(1.71-7.50)	0.001
Sex												
Male	Ref.		Ref.		Ref.		Ref.		Ref.		Ref.	
Female	1.25(1.11-1.41)	<0.001	1.02(0.87-1.19)	0.848	0.97(0.91-1.02)	0.216	0.50(0.40-0.63)	<0.001	0.49(0.36-0.65)	<0.001	0.54(0.37-0.77)	0.001
Route of transmission												
Heterosexual	Ref.		Ref.		Ref.		Ref.		Ref.		Ref.	
Homosexual	1.07(0.96-1.20)	0.226	0.79(0.66-0.94)	0.009	1.03(0.98-1.08)	0.248	0.50(0.37-0.67)	<0.001	0.47(0.31-0.70)	<0.001	0.54(0.35-0.84)	0.006
Others	0.96(0.84-1.10)	0.565	1.82(1.52-2.18)	<0.001	0.85(0.79-0.90)	<0.001	0.65(0.46-0.92)	0.015	0.35(0.19-0.63)	0.001	1.09(0.70-1.71)	0.693
Region												
Northern China	Ref.		Ref.		Ref.		Ref.		Ref.		Ref.	
Northeastern China	1.08(0.94-1.24)	0.253	0.62(0.45-0.85)	0.003	1.20(1.13-1.28)	<0.001	1.51(0.80-2.88)	0.205	0.77(0.31-1.92)	0.57	3.39(1.24-9.30)	0.018
Southern China	1.50(1.30-1.71)	<0.001	0.47(0.35-0.63)	<0.001	1.46(1.37-1.56)	<0.001	0.21(0.07-0.61)	0.005	0.10(0.01-0.78)	0.028	0.26(0.05-1.33)	0.105
Southwestern China	0.43(0.38-0.50)	<0.001	1.99(1.60-2.46)	<0.001	0.54(0.51-0.58)	<0.001	2.73(1.67-4.48)	<0.001	1.86(1.02-3.39)	0.042	4.82(2.00-11.60)	<0.001
Eastern China	0.66(0.58-0.75)	<0.001	0.78(0.61-1.00)	0.05	0.83(0.79-0.88)	<0.001	2.15(1.30-3.57)	0.003	1.20(0.65-2.23)	0.565	4.95(2.05-11.96)	<0.001
Time to ART initiation												
Same-day	Ref.		Ref.		Ref.		Ref.		Ref.		Ref.	

1-7 days	1.03(0.83-1.30)	0.77	0.97(0.68-1.38)	0.864	0.91(0.82-1.00)	0.057	1.23(0.73-2.10)	0.434	0.89(0.46-1.70)	0.72	2.07(0.81-5.29)	0.127
8-30 days	0.96(0.78-1.19)	0.731	0.94(0.68-1.30)	0.689	0.93(0.84-1.02)	0.118	1.38(0.84-2.27)	0.202	1.29(0.72-2.34)	0.393	1.57(0.63-3.89)	0.333
>30 days	0.81(0.65-1.00)	0.051	1.19(0.86-1.64)	0.295	0.83(0.75-0.91)	<0.001	2.21(1.35-3.60)	0.002	1.84(1.02-3.31)	0.042	3.13(1.28-7.66)	0.013
ART year												
2017	Ref.		Ref.		Ref.		Ref.		Ref.		Ref.	
2018	1.66(1.51-1.82)	<0.001	1.20(1.03-1.40)	0.022	1.16(1.12-1.21)	<0.001	1.10(0.90-1.35)	0.334	1.29(0.99-1.68)	0.063	0.91(0.67-1.23)	0.526
2019	3.20(2.78-3.68)	<0.001	2.94(1.65-5.24)	<0.001	1.89(1.79-1.99)	<0.001	2.33(1.53-3.54)	<0.001	4.34(2.52-7.48)	<0.001	1.12(0.56-2.23)	0.749
ART regimen												
NVP	Ref.		Ref.		Ref.		Ref.		Ref.		Ref.	
EFV	0.37(0.32-0.44)	<0.001	0.74(0.56-0.98)	0.037	1.06(0.95-1.17)	0.322	0.64(0.47-0.89)	0.007	0.50(0.35-0.72)	<0.001	1.32(0.64-2.72)	0.456
DTG	1.22(0.98-1.51)	0.078	1.53(0.94-2.48)	0.085	1.58(1.36-1.83)	<0.001	0.56(0.22-1.45)	0.231	0.30(0.07-1.28)	0.103	1.69(0.43-6.61)	0.453
LPV	0.34(0.27-0.42)	<0.001	0.84(0.59-1.20)	0.339	1.29(1.14-1.45)	<0.001	0.53(0.31-0.93)	0.026	0.22(0.09-0.52)	0.001	1.88(0.76-4.63)	0.171
Others	1.51(1.18-1.94)	0.001	0.76(0.34-1.70)	0.502	1.49(1.25-1.78)	<0.001	1.81(0.71-4.63)	0.213	0.73(0.16-3.36)	0.687	6.86(1.83-25.71)	0.004
CD4 at ART initiation (per 100)	0.74(0.72-0.77)	<0.001	1.02(0.98-1.06)	0.413	1.45(1.43-1.47)	<0.001	0.65(0.60-0.70)	<0.001	0.67(0.61-0.74)	<0.001	0.61(0.54-0.68)	<0.001
NRTI backbone												
3TC+TDF	Ref.		Ref.		Ref.		Ref.		Ref.		Ref.	
3TC+AZT	1.01(0.89-1.16)	0.83	1.12(0.90-1.38)	0.309	0.91(0.86-0.96)	0.001	0.75(0.52-1.08)	0.124	0.99(0.65-1.52)	0.978	0.45(0.22-0.91)	0.026
Others	0.68(0.55-0.84)	<0.001	1.94(1.32-2.85)	0.001	0.94(0.83-1.05)	0.282	1.71(0.99-2.95)	0.056	1.94(0.95-3.96)	0.067	1.43(0.62-3.34)	0.403

ART, antiretroviral therapy. LTFU, loss to follow-up. CD4 improvement= transition of people living with HIV from CD4 <500 cells/μL to CD4 >500 cells/μL. NVP, nevirapine. EFV, efavirenz. DTG, dolutegravir. LPV, lopinavir. Others= darunavir, raltegravie, elvitegravir and rilpivirine. aHRs, adjusted hazard ratios. NRTI, nucleotide reverse transcriptase inhibitor. 3TC, lamividine. TDF, tenofovir disproxil fumarate. AZT, zidovudine. Cox model was adjusted for main variables, including baseline age, sex, route of HIV acquisition, region, time to ART initiation, year of ART initiation, CD4+ T-cell counts, and NRTI backbone. 2-sided no adjustment for multiple comparisons.

Table S4 Cox model for the all-cause mortality, AIDS-related mortality, and non-AIDS-related mortality among people living with HIV on ART for each third drug comparison

	All-cause mortality		AIDS-related mortality		Non-AIDS-related mortality	
	aHR (95% CI)	p-value	aHR (95% CI)	p-value	aHR (95% CI)	p-value
EFV vs NVP	0.67(0.48-0.92)	0.014	0.51(0.36-0.74)	<0.001	1.38(0.67-2.87)	0.381
DTG vs NVP	0.33(0.10-1.13)	0.079	0.19(0.03-1.05)	0.057	0.91(0.13-6.42)	0.922
LPV vs NVP	0.48(0.26-0.89)	0.021	0.19(0.07-0.52)	0.001	1.69(0.62-4.58)	0.303
Others vs NVP	2.09(0.44-9.79)	0.351	0.44(0.04-5.26)	0.513	7.63(0.97-60.04)	0.054
DTG vs EFV	0.89(0.35-2.26)	0.813	0.67(0.16-2.85)	0.592	1.18(0.35-4.01)	0.788
LPV vs EFV	0.85(0.53-1.38)	0.518	0.45(0.20-1.04)	0.061	1.46(0.81-2.64)	0.213
Others vs EFV	2.93(1.14-7.55)	0.026	1.46(0.31-6.80)	0.633	6.14(1.79-21.09)	0.004
LPV vs DTG	1.11(0.38-3.20)	0.847	1.04(0.19-5.73)	0.965	1.25(0.33-4.79)	0.74
Others vs DTG	2.72(0.72-10.28)	0.139	2.83(0.32-24.64)	0.347	2.86(0.54-15.02)	0.214
Others vs LPV	2.54(0.83-7.79)	0.102	1.61(0.17-15.65)	0.683	3.06(0.84-11.17)	0.091

ART, antiretroviral therapy. NVP, nevirapine. EFV, efavirenz. DTG, dolutegravir. LPV, lopinavir. Others= darunavir, raltegravie, elvitegravir and rilpivirine. aHRs, adjusted hazard ratios. Poisson regression model with a time offset and robust variances was adjusted for main variables, including baseline age, sex, route of HIV acquisition, region, time to ART initiation, year of ART initiation, CD4+ T-cell counts, and NRTI backbone. NRTI, nucleotide reverse transcriptase inhibitor. 2-sided adjustment for multiple comparisons.

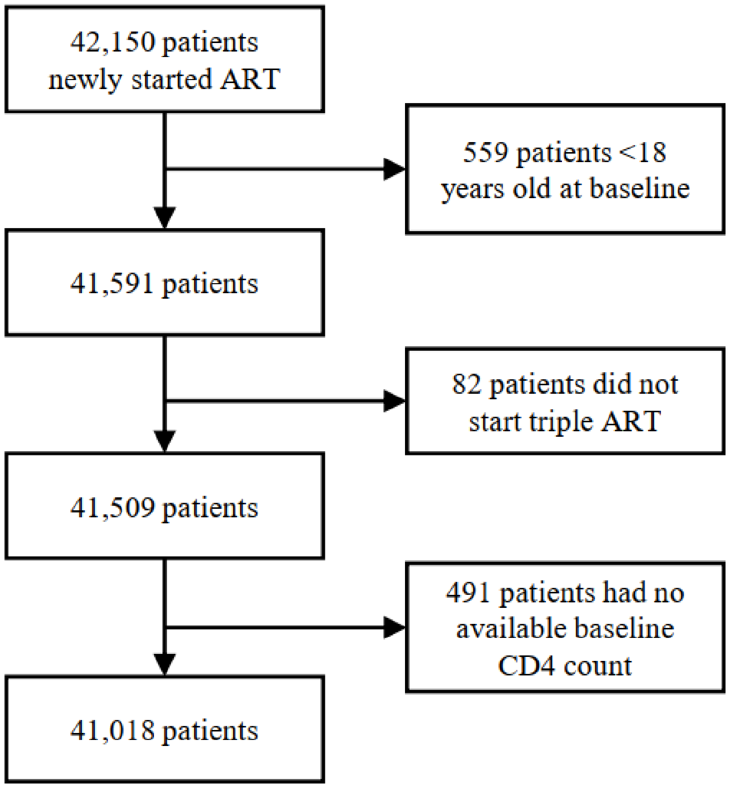

Figure S1 Patient flowchart

ART, antiretroviral therapy.
