# Supplementary material for: Associations of modern initial antiretroviral therapy regimens with all-cause mortality in people living with HIV in resource-limited settings: a retrospective multicenter cohort study in China
Source: Nat Commun. 2023 Sep 2;14:5334. doi: 10.1038/s41467-023-41051-w (PMC10475132; doi:10.1038/s41467-023-41051-w)
Supplement: Supplementary file 3 — Reporting Summary [file 41467_2023_41051_MOESM3_ESM.pdf]

nature portfolio

Corresponding author(s): Gengfeng Fu, Xiaojie Huang, Bin Su, Tong Zhang, Hui Wang, and Huachun Zou

Last updated by author(s): Aug 14, 2023

Reporting Summary

Nature Portfolio wishes to improve the reproducibility of the work that we publish. This form provides structure for consistency and transparency in reporting. For further information on Nature Portfolio policies, see our Editorial Policies and the Editorial Policy Checklist.

Statistics

For all statistical analyses, confirm that the following items are present in the figure legend, table legend, main text, or Methods section.

n/a	Confirmed	
☐	☒	The exact sample size (*n*) for each experimental group/condition, given as a discrete number and unit of measurement
☐	☒	A statement on whether measurements were taken from distinct samples or whether the same sample was measured repeatedly
☐	☒	The statistical test(s) used AND whether they are one- or two-sided<br>*Only common tests should be described solely by name; describe more complex techniques in the Methods section.*
☐	☒	A description of all covariates tested
☐	☒	A description of any assumptions or corrections, such as tests of normality and adjustment for multiple comparisons
☐	☒	A full description of the statistical parameters including central tendency (e.g. means) or other basic estimates (e.g. regression coefficient) AND variation (e.g. standard deviation) or associated estimates of uncertainty (e.g. confidence intervals)
☐	☒	For null hypothesis testing, the test statistic (e.g. *F*, *t*, *r*) with confidence intervals, effect sizes, degrees of freedom and *P* value noted<br>*Give P values as exact values whenever suitable.*
☒	☐	For Bayesian analysis, information on the choice of priors and Markov chain Monte Carlo settings
☒	☐	For hierarchical and complex designs, identification of the appropriate level for tests and full reporting of outcomes
☐	☒	Estimates of effect sizes (e.g. Cohen's *d*, Pearson's *r*), indicating how they were calculated

Our web collection on statistics for biologists contains articles on many of the points above.

Software and code

Policy information about availability of computer code

Data collection	R 4.2.1
Data analysis	R 4.2.1

For manuscripts utilizing custom algorithms or software that are central to the research but not yet described in published literature, software must be made available to editors and reviewers. We strongly encourage code deposition in a community repository (e.g. GitHub). See the Nature Portfolio guidelines for submitting code & software for further information.

Data

Policy information about availability of data

All manuscripts must include a data availability statement. This statement should provide the following information, where applicable:

- Accession codes, unique identifiers, or web links for publicly available datasets
- A description of any restrictions on data availability
- For clinical datasets or third party data, please ensure that the statement adheres to our policy

Source data are provided with this paper. The raw data that support the findings of this study are not publicly available for confidentiality reasons, since these patients may be re-identified through various techniques, such as data linkage or combining datasets. The processed data are available on reasonable request to the corresponding author, HZ, with each request subject to ethical and legislative review from the respective data sources.

Research involving human participants, their data, or biological material

Policy information about studies with human participants or human data. See also policy information about sex, gender (identity/presentation), and sexual orientation and race, ethnicity and racism.

Reporting on sex and gender	Sex was considered in study design, and was determined based on the China ID card system by the NFATP database (with no identifying information available to the researchers). Male: Female= 34709 (84.6%): 6309 (15.4%). All analyses were stratified or adjusted by sex.
Reporting on race, ethnicity, or other socially relevant groupings	Sex and region were social-demographic characteristics, so they were used in our analysis. Sex was determined based on the China ID card system (with no identifying information available to the researchers). Region was determined according to the geographical location of data source (Northern China [Hohhot, Shijiazhuang, Beijing and Tianjin], Northeastern China [Dalian and Shenyang], Southern China [Guangzhou and Shenzhen], Southwestern China [Chongqing and Dehong], and Eastern China [Jiangsu]).
Population characteristics	Age, sex, region were social-demographic characteristics, so they were used in our analysis. Route of transmission, time to ART initiation, CD4 at ART initiation, initial ART regimen, and ART backbone were clinical characteristics, so they were used. Overall, males accounted for 84.6% of the total sample, and the median age was 37 years (interquartile range [IQR] 28-52).
Recruitment	We included all subjects who were older than 18 years, started triple ART between January 1, 2017 and September 30, 2019, and had available baseline CD4+ T-cell count measurements. The follow-up records were collected until study end (December 31, 2019). This may result in underrepresentation of populations that do not meet the criteria (such as children).
Ethics oversight	Ethics approval was obtained from the Ethics Review Committee for Biomedical Research, School of Public Health (Shenzhen), Sun Yat-sen University (Ref: 2020022). The data used in this study do not contain any identifying information available to the researchers.

Note that full information on the approval of the study protocol must also be provided in the manuscript.

Field-specific reporting

Please select the one below that is the best fit for your research. If you are not sure, read the appropriate sections before making your selection.

☒ Life sciences ☐ Behavioural & social sciences ☐ Ecological, evolutionary & environmental sciences

For a reference copy of the document with all sections, see nature.com/documents/nr-reporting-summary-flat.pdf

Life sciences study design

All studies must disclose on these points even when the disclosure is negative.

Sample size	Sample size was not calculated, because the data collectively represent 21.3% of newly reported HIV/AIDS cases in China during this period. We used event per variable (EPV) to assess the data sufficiency. As EPV= 656/9= 72.89>> 10, we confirmed that the sample size was sufficient.
Data exclusions	We included all subjects who were older than 18 years, started triple ART between January 1, 2017 and September 30, 2019, and had available baseline CD4+ T-cell count measurements. Individuals who did not meet the criteria were excluded.
Replication	The analyses in this study were performed by XW under the guidance of Senior Advisor Huachun Zou, and were independently replicated by YC, and YL. The R codes used for statistical analysis and modeling were reviewed by Prof. Junfeng Wang, who specializes in clinical data modeling at Utrecht University in the Netherlands. The analyses were also replicated using Stata 17.0 (another popular software for statistical analysis) by XW under the guidance of Prof. Junfeng Wang and Huachun Zou. All results were verified being reproducible.
Randomization	Randomization was not relevant to the study because it is a cohort study. For all analyses, we fitted an unadjusted model and a model adjusted for the main variables, including baseline age, sex, route of HIV acquisition, region, time to ART initiation, year of ART initiation, CD4 + T-cell counts, and NRTI backbone.
Blinding	Blinding was not relevant to this study because it is a cohort study. Blinding is mainly used in randomized controlled trials (RCTs) to eliminate subjective biases from researchers and participants. However, in cohort studies, researchers do not intervene in the exposure status of participants but rather observe the existing relationship between exposure and outcomes.

Reporting for specific materials, systems and methods

We require information from authors about some types of materials, experimental systems and methods used in many studies. Here, indicate whether each material, system or method listed is relevant to your study. If you are not sure if a list item applies to your research, read the appropriate section before selecting a response.

Materials & experimental systems

n/a	Involved in the study
☒	☐ Antibodies
☒	☐ Eukaryotic cell lines
☒	☐ Palaeontology and archaeology
☒	☐ Animals and other organisms
☒	☐ Clinical data
☒	☐ Dual use research of concern
☒	☐ Plants

Methods

n/a	Involved in the study
☒	☐ ChIP-seq
☒	☐ Flow cytometry
☒	☐ MRI-based neuroimaging
